# BOLD Cerebrovascular Reactivity and NOVA Quantitative MR Angiography in Adult Patients with Moyamoya Vasculopathy Undergoing Cerebral Bypass Surgery

**DOI:** 10.3390/brainsci14080762

**Published:** 2024-07-29

**Authors:** Loris Garbani Nerini, Jacopo Bellomo, Lara Maria Höbner, Vittorio Stumpo, Elisa Colombo, Christiaan Hendrik Bas van Niftrik, Tilman Schubert, Zsolt Kulcsár, Susanne Wegener, Andreas Luft, Luca Regli, Jorn Fierstra, Martina Sebök, Giuseppe Esposito

**Affiliations:** 1 Department of Neurosurgery, University Hospital Zurich, Frauenklinikstrasse 10, 8091 Zurich, Switzerland; loris.garbaninerini@uzh.ch (L.G.N.); jacopo.bellomo@usz.ch (J.B.); lara.hoebner@usz.ch (L.M.H.); vittorio.stumpo@usz.ch (V.S.); elisa.colombo@usz.ch (E.C.); bas.vanniftrik@usz.ch (C.H.B.v.N.); luca.regli@usz.ch (L.R.); jorn.fierstra@usz.ch (J.F.); giuseppe.esposito@usz.ch (G.E.); 2 Clinical Neuroscience Center, University Hospital Zurich, Frauenklinikstrasse 10, 8091 Zurich, Switzerland; tilman.schubert@usz.ch (T.S.); zsolt.kulcsar@usz.ch (Z.K.); susanne.wegener@usz.ch (S.W.); andreas.luft@usz.ch (A.L.); 3 University of Zürich (UZH), Raemistrasse 100, CH-8091 Zurich, Switzerland; 4 Department of Neuroradiology, University Hospital Zurich, Frauenklinikstrasse 10, 8091 Zurich, Switzerland; 5 Department of Neurology, University Hospital Zurich, Frauenklinikstrasse 26, 8091 Zurich, Switzerland

**Keywords:** moyamoya, BOLD-CVR, NOVA, STA-MCA bypass, hemodynamics, quantitative flow

## Abstract

Revascularization surgery for the symptomatic hemisphere with hemodynamic impairment is effective for Moyamoya vasculopathy patients. However, careful patient selection is crucial and ideally supported by advanced quantitative hemodynamic imaging. Recently, blood oxygenation level-dependent cerebrovascular reactivity (BOLD-CVR) and quantitative magnetic resonance angiography with non-invasive optimal vessel analysis (qMRA-NOVA) have gained prominence in assessing these patients. This study aims to present the results of BOLD-CVR and qMRA-NOVA imaging along with the changes in cerebral hemodynamics and flow status following flow augmentation with superficial temporal artery–middle cerebral artery (STA-MCA) bypass in our Moyamoya vasculopathy patient cohort. Symptomatic patients with Moyamoya vasculopathy treated at the Clinical Neuroscience Center of the University Hospital Zurich who underwent hemodynamic and flow imaging (BOLD-CVR and qMRA-NOVA) before and after bypass were included in the analysis. Reduced hemispheric volume flow rates, as well as impaired BOLD-CVR, were measured in all 12 patients with Moyamoya vasculopathy before STA-MCA bypass surgery. Following the surgical procedure, post-operative BOLD-CVR demonstrated a non-significant increase in BOLD-CVR values within the revascularized, symptomatic middle cerebral artery territory and cerebral hemisphere. The results of the statistical tests should be viewed as indicative due to the small sample size. Additionally, post-operative qMRA-NOVA revealed a significant improvement in the hemispheric volume flow rate of the affected hemisphere due to the additional bypass flow rate. Our findings affirm the presence of hemodynamic and flow impairments in the symptomatic hemisphere of the Moyamoya vasculopathy patients. Bypass surgery proves effective in improving both BOLD-CVR impairment and the hemispheric volume flow rate in our patient cohort.

## 1. Introduction

Moyamoya disease is a rare idiopathic vasculopathy characterized by the progressive stenosis or occlusion of the distal internal carotid arteries (ICAs), as well as the proximal anterior (ACAs) and middle cerebral arteries (MCAs), followed by the development of a collateral network of arteries [1,2]. The term “Moyamoya”, derived from Japanese, translates to “puff of smoke”, capturing the extensive cerebral collateral network associated with the disease. The formation of collaterals serves as a compensatory mechanism in response to the decreased cerebral flow caused by the stenosis or occlusion of intracranial arteries. However, this compensatory attempt is often insufficient, resulting in clinical manifestations such as ischemic strokes and transient ischemic attacks (TIAs). Additionally, the dense collateral network tends to cause bleeding, leading to hemorrhagic strokes [1,3].

The treatment objective of symptomatic Moyamoya patients is to enhance cerebral perfusion, thereby reducing the risk for future ischemic and hemorrhagic events [4,5,6]; revascularization surgery via direct, indirect, and combined bypass procedures stands as the sole proven and effective treatment based on clinical trials [7,8,9]. The most recent guidelines on Moyamoya Angiopathy from the European Stroke Organisation (ESO) recommend revascularization surgery using either a direct or a combined bypass procedure [10].

In the historical context, various imaging techniques [11] have been used to assess the vessel status of patients with Moyamoya vasculopathy, with digital subtraction angiography (DSA) remaining a gold-standard technique [12]. However, when analyzing the cerebral perfusion status, techniques such as single-photon emission computed tomography (SPECT) and positron emission tomography (PET) have been utilized. It is noteworthy that these latter methods necessitate exposure to ionizing radiation and have limited availability in routine clinical practice [8,12,13]. Arterial spin labeling is an effective method for detecting the cerebral blood flow in patients with Moyamoya vasculopathy [14].

Recently, two innovative magnetic resonance imaging techniques for the quantitative assessment of the cerebrovascular reserve capacity and blood flow in cerebral vessels have emerged and are increasingly applied in the evaluation of patients with cerebrovascular steno-occlusive disease:

(1) Blood oxygenation level-dependent (BOLD) magnetic resonance imaging (MRI), to evaluate the cerebrovascular reactivity (CVR) at the brain parenchyma level, utilizing the physiological vasodilatory response to CO_2_ [15,16,17];
(2) Quantitative magnetic resonance angiography (qMRA) with non-invasive optimal vessel analysis (NOVA), which measures the volume flow rate (VFR) of large intracranial arteries in mL/min [15,18,19].

The objective of this study is to present the results of BOLD-CVR hemodynamic investigation and qMRA-NOVA flow imaging in our cohort of patients with Moyamoya vasculopathy, along with the changes in cerebral hemodynamics and flow status following revascularization surgery. 

## 2. Methods

### 2.1. Patient Selection

This retrospective cohort study with prospectively collected data includes all adult (>18 years old) patients with symptomatic Moyamoya vasculopathy who underwent a bypass surgery at the Clinical Neuroscience Center of the University Hospital Zurich between May 2019 and May 2023 and who received advanced MR imaging studies (BOLD-CVR and qMRA-NOVA). Following cerebral revascularization, qMRA-NOVA was performed before discharge, and BOLD-CVR was performed 3 months after bypass. All 12 included patients received a combined bypass procedure (direct STA-MCA bypass with indirect synangiosis). The indirect revascularization techniques varied, with 9 patients undergoing encephalo-duro-synangiosis (EDS), 2 patients encephalo-duro-myo-synangiosis (EDMS), and 1 patient encephalo-duro-periosteo-synangiosis (EDPS).

Ethical approval for this study was obtained from the Ethics Committee of the Canton of Zurich under the reference number KEK 2020-02314. All participants provided their consent by signing a general informed consent form. 

### 2.2. Quantitative Advanced MRI Techniques

MRI data were obtained using a 3 Tesla Skyra VD13 system with a 32-channel head matrix following a protocol previously published [20,21]. A (3D) T1-weighted, magnetization-prepared rapid acquisition gradient echo (MP RAGE) image, oriented similarly to the BOLD fMRI scans, was conducted for the purpose of overlay to capture structural information of the entire brain. Additionally, a 3D time-of-flight (TOF) angiography and 2D phase-contrast imaging, utilizing the 3D coordinates determined from the NOVA, were performed to enable VFR analysis [15].

#### 2.2.1. BOLD-CVR Measurement and Analysis

To quantitatively analyze cerebrovascular reactivity at the brain parenchymal level, the BOLD signal was integrated with a standardized vasodilatory CO_2_ stimulus that was achieved through a computer-controlled gas blender employing prospective gas-targeting algorithms (RespirAct^TM^), as per established protocols [22,23]. Following the examination of raw CVR data, this approach yielded quantitative BOLD-CVR values (%BOLD signal change/mmHg CO_2_) assessed for the entire brain, both grey and white matter, and for both hemispheres. Furthermore, employing a vascular atlas on the normalized CVR maps allowed for the calculation of quantitative BOLD-CVR values specific to the vascular territories of both hemispheres, in accordance with previously published methods [15,16].

For this patient cohort, BOLD-CVR values were assessed for the whole brain, as well as for symptomatic and non-symptomatic brain hemispheres, and for symptomatic and non-symptomatic vascular territories (anterior cerebral artery (ACA), middle cerebral artery (MCA), and posterior cerebral artery (PCA) territories).

#### 2.2.2. qMRA NOVA

Volume flow measurements were conducted using the commercially available NOVA (non-invasive optimal vessel analysis) software from VasSol, Inc. Chicago, IL, USA [19]. For the analysis, we focused on the volume flow rates (VFRs) of specific arterial segments: the second segment of the anterior cerebral artery (ACA-A2), the first segment of the middle cerebral artery (MCA-M1), and the second segment of the posterior cerebral artery (PCA-P2). The hemispheric volume flow rate (hVFR) was computed by summing the VFR values for ACA-A2, MCA-M1, and PCA-P2. In the post-bypass qMRA-NOVA analysis, the affected hVFR is calculated as the sum of the VFRs of ACA-A2, MCA-M1, PCA-P2, and the bypass VFR [15,24].

### 2.3. Direct STA-MCA Bypass and Indirect Revascularization

There is no consensus on the optimal type of revascularization surgery. At our institution, we use the STA-MCA bypass as a direct revascularization procedure, connecting the STA to a cortical M4 branch in an end-to-side fashion using microsurgical techniques. Notably, the distal part of the donor STA branch is cut in a fish-mouth fashion to increase its opening diameter, and a linear arteriotomy is performed on the cortical recipient M4 branch, which should be at least 2.5 times the diameter of the recipient. A detailed description of the flow-augmentation bypass procedure is available elsewhere (4). Several indirect techniques have been proposed, evolving over time. We use a combination of vascularized tissue based on patient characteristics and tissue availability, including EDS, EDMS, and EDPS. Combining direct and indirect revascularization procedures leverages the advantages of both techniques [8,25,26].

### 2.4. Statistical Analysis

The statistical analysis was conducted using R. Dichotomous variables are expressed as the frequency (%), while continuous variables are presented as the mean ± standard deviation, and are compared with a paired Student’s *t*-test. A *p* value less than 0.05 was considered statistically significant.

## 3. Results

### 3.1. Study Population Characteristics

Out of the twelve patients included, one individual underwent two bypass operations, initially in one symptomatic hemisphere and, subsequently, after 7 months, in the other meanwhile-symptomatic cerebral hemisphere. For the BOLD-CVR and qMRA-NOVA analyses, each of the two operations was treated separately, resulting in 13 imaging samples. Among the 12 patients who underwent surgery, 9 underwent postoperative qMRA-NOVA analysis, and 11 underwent postoperative BOLD-CVR analysis. In the case of the patient who underwent bypass in both hemispheres, BOLD and NOVA analyses were performed after each operation, leading to 10 qMRA-NOVA and 12 BOLD-CVR imaging samples for the postoperative analysis.

**Figure 1:**
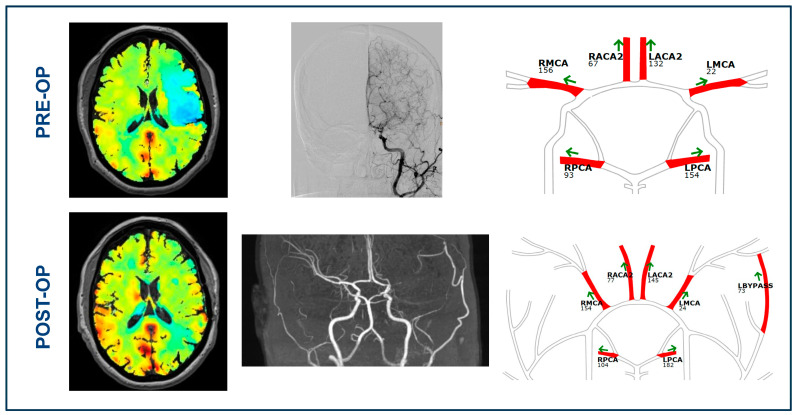
Illustrative case. A 49-year-old patient with Moyamoya vasculopathy presented with bilateral stenosis of the supraclinoid ICA and M1 occlusion on the left side. The patient already experienced two episodes of transitory ischemic attacks in her history, with transient weakness of the right arm and leg, with the last episode occurring two months preoperatively. At the admission, the patient did not have any neurological symptoms. The angiography confirmed the diagnosis and showed a significant presence of collaterals in the left MCA territory. The patient therefore underwent STA-MCA flow-augmentation bypass surgery with encephalo-duro-synangiosis on the left hemisphere. Pre-bypass qMRA NOVA showed an increased flow in the left ACA and PCA, as well as a marked flow reduction in the left MCA. BOLD-CVR showed a steal phenomenon with impaired CVR (hemodynamic failure grade II) for the left MCA territory. After the bypass in the left hemisphere, the qMRA-NOVA showed a patent bypass with a flow of 73 mL/min. The BOLD-CVR showed cortical improvement in the left MCA territory after the bypass operation.

Table 1 presents the baseline characteristics and clinical neurological scores of the included cohort of patients with Moyamoya vasculopathy. Among the included patients, six had unilateral vasculopathy, while the remaining six had bilateral vasculopathy. Three patients (25%) presented with hemorrhagic stroke, four patients (33.3%) with ischemic stroke, and five patients (41.7%) with transient ischemic attacks. Figure 1 illustrates a representative case of a patient with Moyamoya vasculopathy who underwent a surgical revascularization via STA-MCA bypass.

In the case of the patient who underwent bypass in both hemispheres, BOLD and NOVA analyses were performed after each operation, leading to 10 qMRA-NOVA and 12 BOLD-CVR imaging samples for postoperative analysis (Supplementary Table S1).

### 3.2. BOLD-CVR and qMRA-NOVA Imaging Data before Bypass Surgery

Table 2 shows the quantitative hemodynamic BOLD-CVR values, while Table 3 presents the qMRA-NOVA flow values before bypass. When comparing the BOLD-CVR values between the symptomatic and non-symptomatic hemispheres, the symptomatic MCA territory exhibited lower BOLD-CVR values (BOLD-CVR symptomatic MCA territory vs. non-symptomatic MCA territory (%BOLD/mmHgCO_2_): 0.03 ± 0.07 vs. 0.10 ± 0.11; *p* = 0.09), though the difference did not reach statistical significance. The results of the statistical tests should be viewed as indicative due to the small sample size. Similarly, for the ACA territory, the symptomatic territory showed lower absolute values compared to the non-symptomatic one, without a statistically significant difference. A similar trend was noted when comparing the symptomatic and the non-symptomatic hemispheres (BOLD-CVR symptomatic vs. non-symptomatic hemisphere (%BOLD/mmHgCO_2_): 0.09 ± 0.07 vs. 0.12 ± 0.08) (Table 2). 

In the comparison of qMRA-NOVA-derived volume flow rates, a statistically significant difference was observed between the symptomatic and non-symptomatic M1-VFR (symptomatic vs. non-symptomatic (mL/min): 16.15 ± 18.45 vs. 101.38 ± 86.97; *p* < 0.004). Regarding P2-VFR, although no statistically significant difference was found, the symptomatic P2 exhibited a higher flow compared to the non-symptomatic P2 (P2-VFR symptomatic vs. non-symptomatic (mL/min): 164.69 ± 63.20 vs. 123.54 ± 61.59; *p* = 0.10). No difference was observed between symptomatic and non-symptomatic A2-VFRs. The cumulative flow of the symptomatic hemisphere was lower than the flow in the non-symptomatic hemisphere (VFR of symptomatic hemisphere vs. VFR of non-symptomatic hemisphere (mL/min): 282.77 ± 89.70 vs. 322.15 ± 109.05), though this difference did not reach statistical significance (Table 3).

### 3.3. BOLD Cerebrovascular Reactivity after Bypass

**Figure 2:**
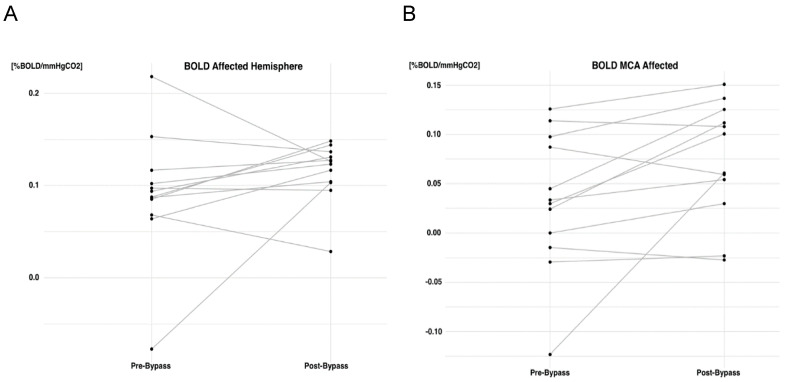
Graphical representation of BOLD-CVR values of the symptomatic (affected); (**A**) hemisphere and MCA. (**B**) territory before and after STA-MCA bypass.

Following cerebrovascular revascularization with STA-MCA bypass, every vascular territory in both the affected and unaffected hemispheres exhibited an improvement in BOLD-CVR, although no statistically significant differences were observed for any (Table 4). The symptomatic MCA territory demonstrated the most significant improvement in BOLD-CVR after bypass surgery (Δ 0.04%BOLD/mmHgCO_2_), followed by the BOLD-CVR improvement of the affected hemisphere (Δ 0.03%BOLD/mmHgCO_2_). Figure 2 illustrates the difference in the BOLD-CVR values of the symptomatic (affected) hemisphere and of the MCA territory before and after bypass revascularization for each patient. Looking at the individual data, we can see that three patients out of twelve showed a decrease in the CVR values of the affected hemisphere following the bypass surgery. All three patients presented with ischemic symptoms, and the bypass was patent following the surgery.

### 3.4. qMRA-NOVA Values after Surgical Revascularization

**Figure 3:**
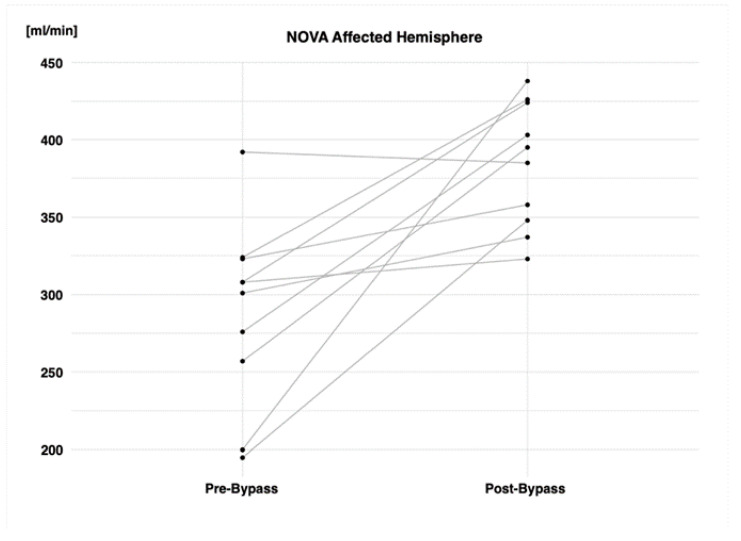
Graphical representation of hemispheric volume flow rates before and after STA-MCA bypass.

Comparing hemispheric pre-bypass and post-bypass volume flow-rate values, a significant improvement after surgical revascularization is noted in the VFRs of the affected hemisphere (affected hemisphere preOP vs. postOP (mL/min): 282.77 ± 89.70 vs. 383.70 ± 40.37; *p* < 0.003). This increase in volume flow rate is attributed to the bypass flow (qMRA-NOVA bypass VFR (mL/min): 86.70 ± 30.32) (Table 5). Figure 3 visually depicts the change in the volume flow rate of the affected hemisphere for each patient before and after the bypass. All but one patient exhibited an increase in the volume flow rate of the affected hemisphere after surgical revascularization. This patient also showed no improvement in the CVR values following revascularization but exhibited the most relevant affected-hemisphere CVR worsening, as shown in the individual data in Figure 2.

## 4. Discussion

Our cohort study revealed a significant improvement in the volume flow rate within the symptomatic hemisphere and a trend towards significant improvement in the BOLD-CVR values of the affected MCA territory, as well as of the affected hemisphere, following combined (direct and indirect) bypass in Moyamoya vasculopathy patients. The improved flow in the affected hemisphere stemmed from the efficacy of the flow-augmentation bypass. The BOLD-CVR analyses demonstrated an interesting trend, with an improvement in the absolute cerebrovascular reactivity values in all vascular territories of the symptomatic hemisphere.

### 4.1. Cerebral Hemodynamics in Patients with Moyamoya Vasculopathy

In individuals with Moyamoya vasculopathy, the gradual narrowing of the supraclinoidal ICA and its proximal branches, coupled with the development of numerous delicate collateral vessels, leads to compromised and often negative cerebrovascular reactivity, elevating the risk of recurrent ischemic stroke events [1,8,27]. The vasodilatation of arterioles and the establishment of collateral flow pathways serve as compensatory mechanisms to maintain regional cerebral blood flow [28,29,30]. Previous studies have utilized impaired BOLD-CVR, along with (recurrent) clinical symptoms, to identify patients who could benefit from surgical revascularization. These studies showed that bypass surgery improved cerebrovascular reactivity in the revascularized hemisphere [31,32,33].

In our cohort, the observed improvement in cerebrovascular reactivity did not reach statistical significance. This can be attributed to three key factors: the timepoint of the advanced neuroimaging, the size of the patient sample, as well as the inclusion of both patients with ischemic and hemorrhagic presentations. In our investigation, which focused on post-operative BOLD-CVR analysis, we considered the initial BOLD-CVR images following surgical revascularization that were obtained approximately three months after the surgery. Given that Moyamoya vasculopathy involves chronic vascular impairment [2], it may take several months for the effects of the surgical revascularization on brain hemodynamics to become more evident. This could explain the lack of significant improvement if the initial post-revascularization BOLD-CVR imaging was conducted “too early” after surgery. However, nine out of twelve patients (75%) showed an improvement in the absolute CVR values of the affected hemisphere and the affected (symptomatic) MCA territory.

Moreover, one important aspect is that the patients underwent a combined (direct + indirect) revascularization. It is known that the impact of revascularization with an indirect bypass promotes neoangiogenesis over time, resulting in more delayed benefits. It could be that the three patients with CVR worsening relied more on the indirect bypass rather than the direct bypass, and therefore, there was no CVR improvement in the first postoperative BOLD-CVR study, since an indirect bypass requires time before its positive effects become apparent. Conversely, in cases where BOLD-CVR images were acquired later after surgical revascularization, an eventual natural progression of Moyamoya disease might lead to a worsening in the hemodynamic status of the contralateral hemisphere, potentially influencing the cerebrovascular reactivity of the revascularized hemisphere. The second factor involves the patient sample. Our study comprised 12 patients, and it is conceivable that a study with a larger cohort could yield statistically significant results.

Our findings align with previous studies, supporting the efficacy of cerebrovascular revascularization surgery in enhancing cerebrovascular reactivity in the affected hemisphere. A study conducted by Han et al. [31] emphasized that extracranial-to-intracranial bypass resulted in a normalized or improved cerebrovascular reactivity in 52 out of 55 impaired hemispheres [31]. Another study by Sam and colleagues demonstrated a post-revascularization improvement in cerebrovascular reactivity in both the affected and unaffected middle cerebral artery territory [34]. In our study, we similarly observed a post-revascularization improvement in the CVR of the affected middle cerebral artery territory. However, we did not observe improvement in the CVR of the unaffected middle cerebral artery territory. Notably, the anterior cerebral artery territory exhibited the lowest improvement in the cerebrovascular reactivity after bypass surgery. This can be attributed to the fact that the STA-MCA bypass directly revascularizes only the MCA territory and does not directly enhance perfusion in the ACA territory. Our results, coupled with the existing literature, affirm that STA-MCA bypass surgery in Moyamoya patients effectively increases the cerebrovascular reactivity and restores cerebrovascular impairment in the middle cerebral artery territory. 

### 4.2. Quantitative Flow Analysis in Patients with Moyamoya Vasculopathy

The preoperative findings from the qMRA-NOVA analysis reveal that the posterior cerebral arteries exhibit the highest VFR values compared to other arteries in the circle of Willis. This observation is attributed to the predominant involvement of stenosis in Moyamoya vasculopathy in the anterior circulation, while the posterior circulation possesses a significant compensatory mechanism due to leptomeningeal collateral engagement. A study conducted by Khan et al. indicates that the flow in the affected posterior circulation decreases six months after STA-MCA bypass surgery [35]. 

The increase in the post-operative volume flow rate in the affected hemisphere is attributed to the STA-MCA bypass, which demonstrated an average flow of 86.70 ± 30.32 mL/min in our study cohort. Notably, two patients exhibited bypass volume flow rates exceeding 100 mL/min (111 mL/min and 152 mL/min, respectively), highlighting the principle that the bypass flow is contingent on the flow demand of the revascularized vascular territory [8,36]. As noted in Table 5, there is a decrease in the mean value of the volume flow rate of the affected M1, which could be due to some backflow caused by strong bypass flow.

### 4.3. Uncovering the Link between Impaired Hemodynamics and Blood Flow in Moyamoya Vasculopathy

Preoperative diagnostic angiography of all 12 enrolled patients with Moyamoya vasculopathy revealed leptomeningeal collaterals in the middle cerebral artery territory originating from both the anterior cerebral artery and posterior cerebral artery. These collaterals serve to compensate for the flow deficit in the middle cerebral artery region. The risk of hemorrhage or ischemic manifestation is contingent upon the efficacy of these collaterals, emphasizing the need to quantify collateral flow [37]. While angiography allows us to demonstrate the cerebrovascular anatomy and to detect the occluded vessels and illustrates the presence of collaterals, it does not assess the impact of collateral vessels on the perfusion of a specific vascular territory, i.e., there is no information about the functionality of these vessels and the flow they provide [27,35,38]. In our study, some patients, despite the bilateral involvement of the supraclinoid ICA and its branches, presented with fewer symptoms compared to other patients with stenosis in a single artery. This once again highlights that angiography alone is insufficient for predicting clinical symptoms (i.e., future stroke events) and functional outcomes in individuals affected by Moyamoya vasculopathy. 

BOLD-CVR and qMRA-NOVA serve as complementary techniques for quantifying the cerebral hemodynamics and flow rate. BOLD-CVR enables the measurement of the cerebrovascular reserve capacity, while qMRA-NOVA provides information about the collateral vessel status and quantitative flow through the intracranial arteries and the bypass [39]. Our BOLD-CVR and qMRA-NOVA values indicated a correlation between impaired BOLD-CVR values and the low-flow areas defined by qMRA-NOVA values. Brain territories exhibiting a lower BOLD-CVR are those perfused by stenotic vessels with a reduced volume flow rate. Abnormal BOLD-CVR values were predominantly observed in the anterior and middle cerebral artery territories, whereas the BOLD-CVR in posterior territories was relatively spared. In all the included patients, the BOLD-CVR of the PCA territory remained preserved. As previously mentioned, this preservation is attributed to the fact that intracranial stenosis in Moyamoya patients primarily involves the anterior circulation, and the posterior circulation possesses a compensatory mechanism, especially through leptomeningeal collaterals [37]. Given that impaired CVR is an important risk factor for future cerebral infarction and can serve as an indication for surgical revascularization [31,40,41], regular monitoring of the cerebrovascular reserve capacity and quantitative blood flow has an important role for patients with Moyamoya vasculopathy. 

### 4.4. Limitations

Moyamoya vasculopathy is relatively rare in Switzerland. Our study specifically focused on the adult population, even though a significant proportion comprises children. The limitations of our study stem from the small size of the patient sample along with the considerable diversity among the included patients (unilateral and bilateral vasculopathy). The indirect revascularization techniques varied among the patients: nine underwent EDS, two underwent EDMS, and one underwent EDPS. These variations in indirect revascularization should not significantly impact hemodynamic and flow outcomes, as indirect bypass takes time to develop. Our BOLD-CVR imaging was conducted around 3 months post-surgery and qMRA-NOVA a few days post-surgery. Additionally, the timing of the advanced quantitative MRI performed after surgical revascularization varied, with NOVA conducted immediately after the surgery and BOLD-CVR conducted approximately 3 months after surgery. 

### 4.5. Future Directions

Our results emphasize the significance of advanced quantitative hemodynamic and flow studies in patients with Moyamoya vasculopathy. Our future objectives involve expanding the patient cohort for analysis, validating our findings in an external cohort, and introducing the BOLD-CVR as a hemodynamic marker and qMRA-NOVA as a flow marker in the decision-making process for determining whether a conservative or surgical treatment is more suitable for individual patients with Moyamoya vasculopathy. Furthermore, our goal is to conduct larger multi-center studies and randomized clinical trials using BOLD-CVR for the hemodynamic assessment of patients with Moyamoya vasculopathy. It is important to note that the BOLD-CVR technique can be easily implemented across different MRI vendors, and the sequence itself takes only a few minutes.

## 5. Conclusions

Our findings affirm the existence of hemodynamic and flow impairments in patients with Moyamoya vasculopathy. Bypass surgery emerges as an effective treatment strategy, demonstrating (non-significant) improvements in impaired BOLD-CVR and an increase in cumulative cerebral blood flow.

## Figures and Tables

**Table 1:** Baseline characteristics of included patient cohort.

	Total Patient Cohort (n = 12)
Age (mean ± SD)	50.25 ± 13.34
Gender: male, n (%)	7 (58.3)
Smoking, n (%)	5 (41.7)
Arterial hypertension, n (%)	3 (25.0)
Dyslipidemia, n (%)	2 (16.7)
Diabetes mellitus, n (%)	4 (33.3)
mRS (median (IQR))	
Before surgery	1 (2)
After surgery	0 (1)
NIHSS (median (IQR))	
Before surgery	0 (2)
After surgery	0 (0)

IQR = interquartile range; mRS = modified Rankin scale; n = number; NIHSS = National Institutes of Health Stroke Scale; SD = standard deviation.

**Table 2:** BOLD-CVR values before the bypass surgery in 12 patients (13 imaging samples).

BOLD-CVR (%BOLD/mmHgCO_2_)	Mean ± SD
CVR whole brain	0.11 ± 0.07
CVR symptomatic hemisphere	0.09 ± 0.07
CVR non-symptomatic hemisphere	0.12 ± 0.08
CVR symptomatic ACA territory	0.08 ± 0.08
CVR non-symptomatic ACA territory	0.11 ± 0.08
CVR symptomatic MCA territory	0.03 ± 0.07
CVR non-symptomatic MCA territory	0.10 ± 0.11
CVR symptomatic PCA territory	0.23 ± 0.06
CVR non-symptomatic PCA territory	0.25 ± 0.06

ACA = anterior cerebral artery; BOLD = blood oxygenation-level dependent; CO_2_ = carbon dioxide; CVR = cerebrovascular reactivity; MCA = middle cerebral artery; PCA = posterior cerebral artery; SD = standard deviation.

**Table 3:** qMRA-NOVA flow values before bypass surgery in 12 patients (13 imaging samples).

qMRA-NOVA (mL/min):	Mean ± SD
VFR symptomatic A2-ACA vessel	96.69 ± 53.94
VFR non-symptomatic A2-ACA vessel	97.23 ± 70.46
VFR symptomatic M1-MCA vessel	16.15 ± 18.45
VFR non-symptomatic M1-MCA vessel	101.38 ± 86.97
VFR symptomatic P2-PCA vessel	164.69 ± 63.20
VFR non-symptomatic P2-PCA vessel	123.54 ± 61.59
VFR symptomatic hemisphere	282.77 ± 89.70
VFR non-symptomatic hemisphere	322.15 ± 109.05

A2 = second segment of the anterior cerebral artery; M1 = first segment of the middle cerebral artery; P2 = second segment of the posterior cerebral artery; VFR = volume flow rate.

**Table 4:** Comparison of pre- and post-bypass BOLD-CVR values in 11 Moyamoya patients (12 imaging series).

BOLD-CVR (%BOLD/mmHgCO_2_)			
(Mean ± SD)	Pre-Bypass (n = 12)	Post-Bypass (n = 12)	*p*-Value
CVR whole brain	0.11 ± 0.07	0.12 ± 0.04	0.52
CVR symptomatic hemisphere	0.09 ± 0.07	0.12 ± 0.03	0.31
CVR non-symptomatic hemisphere	0.12 ± 0.08	0.14 ± 0.06	0.71
CVR ACA symptomatic	0.08 ± 0.08	0.09 ± 0.05	0.62
CVR ACA non-symptomatic	0.11 ± 0.08	0.13 ± 0.06	0.46
CVR MCA symptomatic	0.03 ± 0.07	0.07 ± 0.06	0.09
CVR MCA non-symptomatic	0.10 ± 0.11	0.10 ± 0.09	0.86
CVR PCA symptomatic	0.23 ± 0.06	0.26 ± 0.07	0.29
CVR PCA non-symptomatic	0.25 ± 0.06	0.28 ± 0.10	0.37

ACA = anterior cerebral artery; BOLD = blood oxygenation-level dependent; CO_2_ = carbon dioxide; CVR = cerebrovascular reactivity; MCA = middle cerebral artery; PCA = posterior cerebral artery; SD = standard deviation.

**Table 5:** Comparison of pre- and post-revascularization qMRA-NOVA values.

qMRA-NOVA (mL/min)			
(Mean ± SD)	Pre-Bypass (n = 10)	Post-Bypass (n = 10)	*p*-Value
VFR A2 affected	96.69 ± 53.94	117.10 ± 56.90	0.39
VFR A2 unaffected	97.23 ± 70.46	112.40 ± 60.58	0.59
VFR M1 affected	16.15 ± 18.45	11.00 ± 16.07	0.49
VFR M1 unaffected	101.38 ± 86.97	115.10 ± 111.99	0.74
VFR P2 affected	164.69 ± 63.20	175.40 ± 32.49	0.63
VFR P2 unaffected	123.54 ± 61.59	113.30 ± 32.69	0.64
VFR affected hemisphere *	282.77 ± 89.70	383.70 ± 40.37	0.003
VFR unaffected hemisphere	322.15 ± 109.05	349.00 ± 102.96	0.56
VFR bypass	/	86.70 ± 30.32	/

A2 = second segment of the anterior cerebral artery; M1 = first segment of the middle cerebral artery; P2 = second segment of the posterior cerebral artery; VFR = volume flow rate. * indicates a statistically significant difference between pre- and post-bypass values.

## Data Availability

The original contributions presented in the study are included in the article; further inquiries can be directed to the corresponding author.

## Supplementary Materials

The following supporting information can be downloaded at: https://www.mdpi.com/article/10.3390/brainsci14080762/s1, Table S1: Overview of clinical and quantitative imaging data for all included patients.

## Abbreviations

ACA = anterior cerebral artery; BOLD = blood oxygenation-level dependent; CVR = cerebrovascular reactivity; ICA = internal cerebral artery; M1 = first segment of the middle cerebral artery; MCA = middle cerebral artery; NOVA = non-invasive optimal vessel analysis; qMRA = quantitative magnetic resonance angiography; PCA = posterior cerebral artery; STA = superficial temporal artery.
