# Supplementary material for: BOLD Cerebrovascular Reactivity and NOVA Quantitative MR Angiography in Adult Patients with Moyamoya Vasculopathy Undergoing Cerebral Bypass Surgery
Source: Brain Sci. 2024 Jul 29;14(8):762. doi: 10.3390/brainsci14080762 (PMC11353214; doi:10.3390/brainsci14080762)
Supplement: Supplementary file 1 [file brainsci-14-00762-s001.zip › brainsci-3119785-supplementary.pdf]

Table S1: Overview of clinical and quantitative imaging data for all included patients.

		Case 1		Case 2		Case 3		Case 4		Case 5		Case 6	
**Age (years), sex**		50, M		55, M		51, F		75, M		57, F		43, F	
**Occluded vessel**		R: MCA-M1		R: ICA, ACA-A1, MCA-M1<br>L: ACA-A1		L: MCA-M1		R: MCA-M1, ACA-A1<br>L: MCA-M1		L: ICA, ACA-A1, MCA-M1		R: ICA, MCA-M1, ACA-A1<br>L: ICA, MCA-M1, ACA-A1	
**Clinical presentation**		Multiple episodes Hypesthesia & motor deficits left side		Multiple episodes of frontal headache and dizziness. R Amaurosis fugax		2 Episodes vertigo and R Hemisyndrome		R Hemisyndrome, Aphasia		TIA with Aphasia		TIAs with speech trouble, Aphasia, dysarthria	
**Side of bypass operation**		R		R		L		L		L		L	
**Indirect bypass technique**		EDS		EDS		EDS		EDMS		EDS		EDPS	
**Smoking**		No		Yes		No		No		Yes		No	
**Hypertension**		No		Yes		Yes		No		No		No	
**Dyslipedemia**		No		No		No		No		Yes		No	
**Diabetes mellitus**		No		Yes		Yes		No		No		No	
**Presentation**		Unilateral, TIAs		Bilateral, Ischemic		Unilateral, TIAs		Bilateral, hemorrhagic		Unilateral, TIA		Bilateral, TIAs	
**NIHSS score (pre- / post- surgery)**		0	0	0	0	0	0	4	0	0	0	0	0
**mRS score (pre- / post -surgery)**		0	0	0	0	1	0	2	3	1	0	1	3
**Time between surgery and post-surgery qMRA-NOVA (days)**		1		1		2		NA		3		NA	
**Time between surgery and post-surgery BOLD-CVR (days)**		80		145		109		181		87		2	
**qMRA-NOVA (pre- / post-surgery)**	**affected A2-flow**	183	175	89	96	132	145	0	/	84	59	19	/
	**unaffected A2-flow**	128	140	13	21	67	77	0	/	66	91	27	/
	**affected M1-flow**	3	0	44	0	22	24	0	/	7	0	44	/
	**unaffected M1-flow**	186	173	163	214	161	154	0	/	128	218	50	/
	**affected P2-flow**	206	166	191	219	154	182	98	/	166	239	179	/
	**unaffected P2-flow**	76	85	72	111	92	104	152	/	62	73	176	/
	**affected hemispheric VFR**	392	385	324	426	308	424	98	/	257	395	242	/
	**unaffected hemispheric VFR**	390	398	248	346	320	335	152	/	256	382	253	/
	**Bypass flow**	44		111		73		/		97		/	
**BOLD-CVR (pre- / post-surgery)**	**affected ACA territory**	0.187	0.131	0.1274	0.1304	0.0708	0.0758	0.1743	0.1243	0.0564	0.0297	0.0148	-0.0195
	**unaffected ACA territory**	0.2004	0.14	0.1562	0.1585	0.0803	0.0616	0.1086	0.156	0.143	0.1724	0.0215	-0.0047
	**affected MCA territory**	0.0872	0.0594	0.1139	0.1081	0.0335	0.0542	0.1259	0.1509	-0.0146	-0.0273	-0.0294	-0.0231
	**unaffected MCA territory**	0.2476	0.1588	0.2261	0.2203	0.1032	0.0588	0.1349	0.1527	0.1312	0.1662	-0.0132	0.0038
	**affected PCA territory**	0.3006	0.2195	0.3028	0.2769	0.182	0.1777	0.1464	0.3846	0.2387	0.3072	0.2435	0.2392
	**unaffected PCA territory**	0.2761	0.1877	0.2837	0.257	0.1626	0.1684	0.1809	0.5097	0.2601	0.3297	0.3006	0.2108
	**affected hemisphere**	0.2182	0.1264	0.1532	0.1367	0.0871	0.1042	0.1021	0.1232	0.0972	0.0948	0.0683	0.0285
	**unaffected hemisphere**	0.2655	0.1638	0.1882	0.1882	0.105	0.0552	0.0793	0.1195	0.1557	0.1975	0.0581	0.0693

		Case 7		Case 8		Case 9		Case 10		Case 11		Case 12		
**Age (years), sex**		69, M		39, M		41, M		36, F		30, F		57, M		
**Occluded vessel**		R: MCA-M1<br>L: MCA-M1		R: MCA-M1, ACA-A1<br>L: MCA-M1, ACA-A1		R: ICA		L: ICA, MCA-M1		L: ICA, MCA-M1		R: ICA, MCA-M1<br>L: MCA-M1		
**Clinical presentation**		Homonym hemianopsia left, paresthesia R+L,		R Hemisyndrome		Forgetfulness, concentration		Headache, homonym hemianopsia		Hemiparesis R, speech trouble		R hemisyndrome, Aphasia		
**Side of bypass operation**		L		L		R		L		L		L		
**Indirect bypass technique**		EDS		EDS		EDMS		EDS		EDS		EDS		
**Smoking**		Yes		Yes		No		No		Yes		No		
**Hypertension**		Yes		No		No		No		No		No		
**Dyslipedemia**		Yes		No		No		No		No		No		
**Diabetes mellitus**		Yes		No		Yes		No		No		No		
**Presentation**		Bilateral, Ischemic		Bilateral, hemorrhagic		Unilateral, TIAs		Unilateral, hemorrhagic		Unilateral, Ischemic		Bilateral, Ischemic		
**NIHSS score (pre- / post- surgery)**		2	0	3	1	0	0	0	0	0	0	2	1	1
**mRS score (pre- / post- surgery)**		2	0	3	1	1	1	0	0	0	0	4	2	1
**Time between surgery and post-surgery qMRA-NOVA (days)**		2		3		2		1		NA		1<br>1		
**Time between surgery and post-surgery BOLD-CVR (days)**		61		99		NA		56		72		187<br>115		
**qMRA-NOVA (pre- / post- surgery)**	**affected A2-flow**	112	194	29	8	148	157	103	98	149	/	116	93	146
	**unaffected A2-flow**	124	106	66	78	112	110	76	91	182	/	157	246	164
	**affected M1-flow**	0	11	0	0	0	0	0	0	15	/	41	34	41
	**unaffected M1-flow**	10	0	0	0	158	48	229	309	201	/	14	18	17
	**affected P2-flow**	88	176	272	177	128	156	92	157	288	/	151	128	154
	**unaffected P2-flow**	77	121	248	150	53	60	85	145	201	/	160	152	132
	**affected hemispheric VFR**	200	438	301	337	276	403	195	348	452	/	308	323	358
	**unaffected hemispheric VFR**	211	227	314	228	323	218	390	545	584	/	331	416	395
	**Bypass flow**	57		152		90		93		/		68	82	
**BOLD-CVR (pre- / post- surgery)**	**affected ACA territory**	-0.0749	0.0754	0.0745	0.1307	0.1732	/	0.0651	0.1176	-0.003	0.0675	0.0692	0.1285	0.1467
	**unaffected ACA territory**	-0.1022	0.0778	0.0858	0.1288	0.1572	/	0.1176	0.144	0.1676	0.2444	0.0842	0.1634	0.0945
	**affected MCA territory**	-0.1231	0.0608	0.0977	0.1367	-0.0105	/	0.0449	0.1254	0.0243	0.1118	0.0001	0.0298	0.1006
	**unaffected MCA territory**	-0.1766	-0.093	0.0575	0.1163	0.2083	/	0.1265	0.1538	0.1436	0.2016	0.0061	0.0481	0.047
	**affected PCA territory**	0.1707	0.2361	0.214	0.2807	0.3662	/	0.1781	0.178	0.2618	0.384	0.1909	0.2266	0.2202
	**unaffected PCA territory**	0.166	0.3045	0.2341	0.304	0.3804	/	0.218	0.213	0.2939	0.4335	0.224	0.2865	0.1819
	**affected hemisphere**	-0.0773	0.1032	0.0938	0.131	0.1219	/	0.0852	0.1484	0.0875	0.1441	0.0641	0.1166	0.1274
	**unaffected hemisphere**	-0.0444	0.0558	0.0878	0.1246	0.1922	/	0.1282	0.1594	0.1873	0.2665	0.0889	0.129	0.0961

Abbreviations: ACA=anterior cerebral artery, A1=first segment of anterior cerebral artery, A2=second segment of anterior cerebral artery, BOLD=blood oxygenation level-dependent, CVR=cerebrovascular reactivity, EDS=encephalo-duro-synangiosis, EDMS=encephalo-duro-myo-synangiosis, EDPS=encephalo-duro-periosteo-synangiosis, F=female, ICA=internal cerebral artery, L=left, M=male, MCA=middle cerebral artery, M1=first segment of middle cerebral artery, mRS=modified Rankin Scale, NIHSS=National Institutes of Health Stroke Scale, NOVA=non-invasive vessel analysis, PCA=posterior cerebral artery, P2=second segment of posterior cerebral artery, qMRA=quantitative magnetic resonance angiography, R=right, TIA=transient ischemic attack, VFR=volume flow rate
